# Independent Treatment Centres Are Not a Guarantee for High Quality and Low Healthcare Prices in The Netherlands – A Study of 5 Elective Surgeries

**DOI:** 10.15171/ijhpm.2019.144

**Published:** 2020-01-07

**Authors:** Anouk Dorine Maria Tulp, Florien Margareth Kruse, Niek Waltherus Stadhouders, Patrick P.T. Jeurissen

**Affiliations:** ^1^IQ healthcare, Radboud University and Medical Center, Nijmegen, The Netherlands.; ^2^Ministry of Health, Welfare and Sport, The Hague, The Netherlands.

**Keywords:** Independent Treatment Centres, Focus Factory Theory, Ambulatory Care, Quality of Care, The Netherlands

## Abstract

**Background:** Independent treatment centres (ITCs) are a growing phenomenon in many healthcare systems. Focus factory theory predicts that ITCs provide high quality healthcare with low prices, through specialisation, high-volume and routine. This study examines if ITC care outperforms general hospital (GH) care within a regulated competition system in the Netherlands, by focusing on differences in healthcare quality and price.

**Methods:** The cross-sectional study combined publicly available quality data, list prices and insurer contracts for 2017. Clinical outcomes of 5 elective surgeries (total hip and knee replacement, anterior cruciate ligament (ACL), cataract and carpal tunnel surgeries) were compared using zero-or-one inflated beta-regressions, corrected for underlying structural factors (ie, volume of care, process and structure indicators, and chain affiliation). Furthermore, price differences between ITCs and GHs were examined using ordinary least squares regressions. Lastly, we analysed quality of care in relation to the number of insurance contracts of the 4 largest Dutch insurance companies using ordered logistic regressions.

**Results:** Quality differences between ITCs and GHs were found to be inconsistent across procedures. No facility type performed better overall. There were no differences exhibited in the list prices between ITCs and GHs. No consistent relationship was found between the underlying factors and quality or price, in different procedures and time. We found no indication for selective contracting based on quality within the ITC sector.

**Conclusions:** This study found no evidence that ITCs outperform GHs on quality or price. This evidence does not support the focus factory theory. The substantial practice variation in quality of care may justify more evidence-based contracting within the market for elective surgery.

## Background

Healthcare systems worldwide strive to improve the quality of care, while experiencing a growing need to curb the increasing healthcare costs.^1^ As a response, governments aim to improve quality and reduce costs simultaneously.^2,3^ One of the proposed solutions is the reallocation of ambulatory care from general hospitals (GHs) to independent treatment centres (ITCs).^4,5^

ITCs are a growing phenomenon in many healthcare systems. In the United Kingdom, the number of ITCs grew from 10 in 2006-2007 to 161 ITCs in 2010-2011.^6,7^ In the Netherlands, the number of ITCs increased by 87%, from 229 ITC sites in 2009 to 418 ITC sites in 2016, while the number of invasive treatments performed in ITCs nearly tripled.^8^ Yet, the share of ITCs within total reimbursable healthcare in the Netherlands is only 3.8% in 2016.^9^ The expansion of ITCs may be explained by increased possibilities to perform more invasive procedures in outpatient settings, as a result of technological developments.^10^ Due to the increasing significance of ITC care, it is important to study cost- and quality differences between ITC care and GH care, and investigate how this is supported within a regulated competition healthcare system.

The Dutch healthcare system regulates ITCs and GHs to a great extent. Healthcare providers that provide reimbursable medical care are not allowed to allocate profits to third parties. Hence, ITCs offering reimbursable care are non-profit entities, as are hospitals.The classification of ITCs as a distinct type of healthcare provider was formalised in 1998, when ITCs were allowed to provide reimbursable medical care for a limited array of treatments. The rationale behind the legislation was to reduce waiting lists and to control for-profit clinics.^11,12^ In 2005, a formal distinction between ITCs and hospitals was abolished with the introduction of the Health Care Institutions Admission Act. This act classifies hospitals and ITCs equally as medical specialist care providers. However, in practice, ITCs differ significantly from hospitals in their organisational set-up: ITCs are much smaller, offer primarily elective ambulatory care, and tend to be more focused.^13^ In practice, ITCs are still categorised differently by various stakeholders in the Dutch healthcare system.

The Dutch healthcare system was reformed in 2006. Since then, consumers have been able to freely choose their health insurers and healthcare providers.^14,15^ In this regulated competition system, health insurers purchase healthcare selectively from different providers, and negotiate features, such as volume, price and quality. Insurers offer 2 types of plans: a benefits in-kind plan and a restitution plan.^16^ A restitution plan reimburses all providers, guaranteeing full choice for consumers (± 20% of the population). A benefits in-kind plan offers full reimbursement to a restricted network of providers, and partial reimbursement (usually 75%) to out-of-network providers.^17^

Patients seeking care at ITCs are likely to differ from those visiting GHs.^13^ For example, patients have different pre-requisites or preferences in choosing between GHs and ITCs. The location and presence of certain physicians are important factors in patients’ choice of GHs, whereas quality of care and limited waiting time are important motivations for patients opting for ITCs.^18^ Important information sources for patients choosing an ITC include advice from friends and acquaintances (47%), and the internet (42%). Information for choice of GH is often gained from previous experiences (57%) or advice from a general practitioner or physician (30%).^18^

### Theory

ITCs often specialise in a specific set of elective low-invasive medical procedures.^11^ Their concept originates from the ‘focus factory’ theory, which builds on specialisation – with the intention to yield benefits from simplicity, repetition, experience and homogeneity of performances. This theory implies increased productivity and quality improvements as a result of focus.^13,19^ Thus, the focus and narrow scope of ITCs might lead to better performances, compared to GHs.

Healthcare performance could be driven by a number of underlying factors related to the focus factory theory. The focus of ITCs could be reflected in improved performance on process and structure indicators, due to standardisation and improved coordination of processes.^20^ This might also reduce overhead costs, leading to lower production costs and potentially lower prices. Moreover, high volume could improve quality – known as the volume-quality relationship.^21^ Furthermore, chain membership (ie, facilities with multiple sites) could improve quality through the benefits of greater resources and organisational knowledge from other chain members.

Selective contracting of efficient and effective care by health insurers could be an important driver to improve quality within the ITC sector. Almost every hospital in the Netherlands is contracted by the main insurance companies, but this is not the case for ITCs.^22^ ITCs might, therefore, feel inclined to profile themselves as a provider of high quality care with low prices, to compete with hospitals that have greater market power. Representatives of the ITC sector deemed this pressure as very high, as they experience difficulties in obtaining contracts from health insurers.^22^

The aim of this study is to compare ITCs to GHs on quality of care and price. The main research questions are: Do quality outcomes differ between ITCs and GHs? Do prices differ between ITCs and GHs? Furthermore, 2 supporting research questions were asked towards understanding the determinants behind potential performance differences. (*i*) Which underlying factors are associated with quality outcomes or prices? (*ii*) Is selective contracting within the ITC sector based on quality outcomes of the previous year?

## Methods

### Data and Variables

Quality data of Dutch hospitals and ITCs for 2017 was extracted from the public dataset of the Dutch National Health Care Institute (Zorginstituut Nederland). Five medical procedures were selected based on the availability of clinical outcome data: anterior cruciate ligament (ACL) surgery, cataract surgery, total hip replacement (THR), total knee replacement (TKR), and carpal tunnel syndrome (CTS) surgery. We collected (1) clinical outcomes, (2) process and structure indicators, and (3) the annual number of surgeries per facility. The quality indicators were selected and defined by various stakeholders (eg, the respective medical specialist associations); the Dutch National Health Care Institute and the Dutch Health Care Authority (NZa) facilitated and managed this process. Percentage postoperative infections after CTS and the percentage of revisions after TKR, THR, and ACL surgery were used as clinical outcomes. The quality indicators for CTS, TKR, THR, and ACL were negatively framed (which means that high quality was represented by a percentage close to zero). Postoperative improved visual acuity (ie, ≥1 line improved on eye chart) and comparisons between achieved refraction and target refraction were used as clinical outcome measures for cataract surgery, and were positively framed (which means that high quality was represented by a percentage close to 100). Patients with ocular comorbidities were excluded from the quality dataset for cataract surgery. THR or TKR revision percentages were case-mix adjusted (ie, gender, age, ASA classification, diagnosis, body mass index, Charnley classification and smoking) by the Dutch Arthroplasty Register.^23-25^ For CTS and ACL surgery, no data on case-mix was available.

An index measure was made upon the various process and structure indicators for each individual medical procedure. For example, if a facility uses a decision aid, the process measure will be 1 (good performance). If a facility does not comply to this standard, the process measure will be 0 (poor performance). The dichotomous quality indicators (see Supplementary file 1, Table S1) were transformed into z-scores.^26,27^ Index measures were based upon the mean of the z-scores of the individual quality indicators. No index was constructed for ACL treatments due to the absence of process and structure indicators.

To collect price data, we first selected the most frequently used surgical diagnostic related groups ([DRGs] referred to as DOTs in Dutch) per treatment. In the Netherlands, prices are freely negotiable: each insurer and provider negotiate a DRG-price for contracted care. As these prices are competition-sensitive, they are not made public, and were not available for this study. However, providers are legally obliged to publish list prices. In theory, these prices apply for patients without health insurance or patients who receive care from a non-contracted provider. When patients visit out-of-network-providers, they may pay up to 25% of the list price, out-of-pocket. Therefore, list prices may be used as a proxy for contracted prices, although list prices are generally higher than contracted prices. List prices of the first quarter of 2017 were obtained by manually searching websites and directly contacting healthcare providers during December 2018.

A dichotomous variable for chain affiliation was constructed manually. Providers with at least 2 sites (ie, different unique addresses) were categorised as chains. In order to also include outpatient hospital clinics, a dataset from the Dutch Ministry of Health, Welfare of Sport was used.^28^

Data on whether or not insurance companies contracted ITCs for the 5 medical procedures were obtained by hand-searching the websites of the 4 largest insurance companies (CZ, Zilveren Kruis, VGZ, and Menzis, which together covered 88.4% of the Dutch insurance market in 2017^29^) in December 2018. The list of providers that were contracted in each limited provider plan was used to construct the total number of contracts per ITC.

Before the analysis was performed, this study imposed several restrictions to the data. We had to exclude healthcare providers that did not provide annual quality data. These consist of: 4 ITC observations and 1 GH observation for cataract surgery; 4 ITC observations and 1 GH observation for CTS; 4 ITC observations for THR and TKR; and 4 ITC observations and 2 GH observations for ACL. Furthermore, 5 facilities were not able to deliver list prices for 2017, and were excluded from the price analyses. We excluded specialty and academic hospitals from all analyses, because they tend to treat a different and more complex patient group compared to ITCs and GHs.^30^ In addition, specialty and academic hospitals have teaching obligations that could affect quality and price. This assumption was relaxed in the robustness analyses. We also excluded providers that delivered data as holding companies only. This means that we had to remove those providers that provided the same care at multiple sites, but the different sites did not report their individual data. This resulted in the exclusion of 1 ITC chain that provided care for all 5 medical procedures included in this study. To identify observations with a potentially great influence on the regression coefficients, we performed Cook’s distance tests on all regression models.^31^ Since our sample size was relatively small and single infections could lead to high infection percentages in providers with low volumes; our results could have been driven by outliers. A Cook’s distance value >0.85 was required for an observation to be considered influential.^32^ This resulted in exclusion from the regression analysis. One TKR observation and one ACL observation were identified as highly influential. Furthermore, ordinal logistic regression was only performed with sufficient sample size (n > 10), therefore, no models on insurance contracts were conducted on THR (n = 9) and TKR (n = 10).

### Data Analysis

For each medical procedure, 5 regressions were run; 3 models with quality as a dependent variable, one with list price as a dependent variable and one with the number of insurance contracts as a dependent variable. In all regression models, standard errors were clustered on chain affiliation level.

Firstly, we tested for differences in clinical outcomes between ITCs and GHs (Table S2 Model 1a). Secondly, we checked which underlying factors might drive clinical outcomes (ie, volume, process and structure measures, and chain affiliation) (Table S2 Model 1b). Thirdly, we combined these 2 models to assess if the relationship between the type of provider and clinical outcomes persists when controlling for underlying factors (Model 1). As the outcome measures are bounded by 0% and 100%, with a significant portion of the observations at the extremes, zero-or-one inflated beta regressions were used.^33,34^ In these models, coefficients should be interpreted as elasticities. The marginal effects were calculated separately through the margins command in Stata 15^®^ and reported in the text. Fourthly, an ordinary least squares model was applied to identify differences in list price between ITCs and GHs, while correcting for underlying factors (volume, process and structure measures, and chain affiliation) (Model 2). (In Figure S1, we display the residual plots to assess if the residuals after the ordinary least squares regressions are normally distributed. Please note that we had already clustered our observations within chains to limit this possibility. These plots illustrate that no irregular variances of residuals can be detected.) Fifthly, by means of an ordered logistic regression, the number of contracted ITCs in 2018 was related to quality (ie, clinical outcomes) in the previous year (Model 3). We used an ordered logistic regression, as the dependent variable – number of contracts – should be treated as ordered categorical classes.

We performed several robustness checks. Firstly, we repeated the analyses with quality data from 2016. No quality data of previous years could be used, as different quality indicators were used prior to 2016. Secondly, we estimated if the exclusion of specialist and academic hospitals had a significant impact on the result. Thirdly, we checked if results changed when outliers were included (ie, 1 TKR observations and 1 ACL observation).

## Results

### Descriptive Statistics

Summary statistics are given in Table 1. Quality differences are small and inconsistent; ITCs outperform GHs on cataract care, CTS and ACL surgery, but perform on average worse on THR and TKR. However, standard errors are often large, indicating high variation in quality outcomes in both ITCs and GHs. All procedures except ACL surgery are performed on average more frequently in GHs. The majority of ITCs are affiliated to a chain (50%-62%), with chain affiliation rates being especially high for ITCs performing TKR and THR (respectively 60% and 62%). Most GHs are affiliated to a chain (ie, having at least 2 sites – including outpatient clinics) as well (55%-57%). GHs perform better on process and structure indicators (Table S1), as illustrated by the average index measure being negative for ITCs and positive for GHs.

**Table 1:** Descriptive Statistics 2017

	**Cataract**		**CTS**		**TKR**		**THR**		**ACL**	
	**ITC**	**GH**	**ITC**	**GH**	**ITC**	**GH**	**ITC**	**GH**	**ITC**	**GH**
**Quality Indicators**										
Postoperative ≤1 dioptre of target refraction [%]	94.87 ± 3.32 (30)	93.78 ± 3.45 (64)								
Postoperative improved visual acuity ≥1 line [%]	85.58 ± 9.81 (30)	83.10 ± 7.25 (64)								
Postoperative infectionwithin 30 days [%]			0.15 ± 0.31 (20)	0.28 ± 0.46 (69)						
Revision within 1 year [%]					2.72 ± 3.29 (10)	1.28 ± 0.89 (69)	1.93 ± 2.06 (9)	1.69 ± 1.06 (69)	2.92 ± 5.73 (14)	3.75 ± 2.75 (66)
Process and structure measure^a^ [index]	-0.12 ± 0.38 (32)	0.08 ± 0.35 (65)	-0.13 ± 0.64 (24)	0.05 ± 0.39 (70)	-0.68 ± 0.55 (15)	0.19 ± 0.47 (69)	-0.39 ± 0.65 (13)	0.13 ± 0.57 (69)		
**Volume Indicators**										
Surgeries [n]	1180.81 ± 640.65 (31)	1855.22 ± 965.50 (65)	118.05 ± 132.30 (22)	369.99 ± 199.76 (69)	163.07 ± 182.84 (15)	315.00 ± 149.90 (69)	127.92 ± 130.68 (13)	379.51 ± 184.63 (69)	129.22 ± 139.31 (18)	78.84 ± 66.08 (68)
Chain affiliation [dummy]	0.50 ± 0.51 (32)	0.57 ± 0.50 (65)	0.50 ± 0.51 (24)	0.56 ± 0.50 (70)	0.60 ± 0.51 (15)	0.55 ± 0.50 (69)	0.62 ± 0.51 (13)	0.55 ± 0.50 (69)	0.56 ± 0.51 (18)	0.56 ± 0.50 (68)
**Prices and Contracts**										
Insurance contracts [n]	3.44 ± 1.11 (32)	3.94 ± 0.24 (64)	2.96 ± 1.27 (24)	3.91 ± 0.28 (69)	2.60 ± 1.55 (15)	3.96 ± 0.21 (68)	2.62 ± 1.61 (13)	3.96 ± 0.21 (68)	2.94 ± 1.35 (18)	3.94 ± 0.30 (67)
List price surgery^b^ [€]	1230.37 ± 116.00 (31)	1235.89 ± 212.94 (64)	998.03 ± 180.81 (20)	926.11 ± 215.33 (69)	10 402.41 ± 1115.47(14)	10 079.14 ± 920.37 (68)	9905.91 ± 1125.74 (12)	9344.06 ± 887.66 (68)	4208.94 ± 425.02 (14)	4243.23 ± 726.79 (67)

Abbreviations: GH, general hospital; ITC, independent treatment centre; THR, total hip replacement; TKR, total knee replacement; CTS, carpal tunnel syndrome; ACL, anterior cruciate ligament. Values are presented as mean ± standard error (n). ^a^See Table S1 for overview. ^b^The following DRG codes were used: Cataract surgery - 070401008; CTS - 990004071; TKR - 131999104; THR - 131999052; ACL – 131999102.

Average list prices are higher in ITCs for TKR, THR and CTS surgeries, but lower for cataract and ACL surgeries. The variance in surgery list prices on TKR and THR is larger within ITCs. Furthermore, the vast majority of GHs are contracted by the 4 largest insurance companies (on average 3.91-3.96). The ITCs are contracted substantially less and with greater variance (on average 2.60-3.44). All GHs have insurance contracts with at least one of the 4 insurance companies, which is not the case for all ITCs.

### Regression Analyses

No clear quality differences were found between ITCs and GHs for TKR, cataract and CTS surgeries (Table 2 and Table S2). Model 1a (Table S2) estimates that ITCs have a higher revision rate for THR, but a lower revision rate for ACL. Both relationships persist when correcting for underlying factors (Table 2). The estimated elasticity of 0.82 for THR translates into a 1.44 percentage point higher revision rate in ITCs. ITCs performed 2.21 percentage point fewer revision surgeries than GHs for ACL. Table 2 indicates that the chance of developing postoperative infections declines when more CTS surgeries are performed. However, a volume-quality relationship was not found for any of the other procedures. Similarly, the process and structure indicators are only related to one procedure: they are positively associated with the increase of postoperative dioptre of target for cataract care. Chain affiliation seems unrelated to quality.

**Table 2:** Zero-or-One Inflated Beta Regression Models on Quality in Relation to Facility Type (ie, ITC Versus GH) 2017

**Dependent Variable**	**Cataract** ^a^		**CTS**	**TKR**	**THR**	**ACL**
	**Postoperative ≤1 Dioptre of Target [0-1]**	**Improved Visual Acuity ≥1 line [0-1]**	**Postoperative Infection [0-1]**	**Revision Within 1 Year [0-1]**	**Revision Within 1 Year [0-1]**	**Revision Within 1 Year [0-1]**
**Model 1**	n = 94	n = 94	n = 89	n = 78	n = 78	n = 79
GH	*Reference*	*Reference*	*Reference*	*Reference*	*Reference*	*Reference*
ITC	0.20 ± 0.14	0.09 ± 0.14	-0.08 ± 0.17	0.62 ± 0.45	0.82***± 0.20	-0.69**± 0.29
No. of surgeries (x100)	-0.00 ± 0.01	-0.00 ± 0.01	-0.14** ± 0.07	-0.07 ± 0.04	-0.02 ± 0.04	0.04 ± 0.11
Process/structure	0.49** ± 0.23	-0.13 ± 0.25	-0.26 ± 0.14	0.02 ± 0.17	0.10 ± 0.11	
No chain affiliation	*Reference*	*Reference*	*Reference*	*Reference*	*Reference*	*Reference*
Chain affiliation	-0.08 ± 0.12	-0.25 ± 0.16	0.34 ± 0.18	0.10 ± 0.16	0.09 ± 0.13	-0.08 ± 0.20

Abbreviations: GH, general hospital; ITC, independent treatment centre; THR, total hip replacement; TKR, total knee replacement; CTS, carpal tunnel syndrome; ACL, anterior cruciate ligament. Values are presented as coefficient ± clustered standard error. ^a^The dependent variables of the cataract models are positively framed (one-inflated beta regressions), where the others are negatively framed (zero-or-one inflated beta regressions). *** *P*  < .01, ** *P*  < .05.

#### Price and Facility Type

The association between list prices and facility type is shown in Table 3. No differences in list prices were found between ITCs and GHs after correction for additional factors. High volume is related to a lower list price for standard cataract surgery, although the effect is limited: each additional surgery lowers the list price by approximately €0.05. Furthermore, good performances on process and structure measures are related to higher surgery prices for CTS surgery. This means that one standard-deviation increase in process and structure indicators increases list prices by €121.

**Table 3:** Relation Price and Facility Type (ie, ITC Versus GH) 2017

**Dependent Variable**	**Cataract**	**CTS**	**TKR**	**THR**	**ACL**
	**List Price Surgery [€]**	**List Price Surgery [€]**	**List Price Surgery [€]**	**List Price Surgery [€]**	**List Price Surgery [€]**
**Model 2**	n = 94	n = 87	n = 82	n = 80	n = 81
GH	*Reference*	*Reference*	*Reference*	*Reference*	*Reference*
ITC	-49.15 ± 41.61	51.62 ± 56.59	203.98 ± 519.83	460.97 ± 465.21	-2.98 ± 160.13
No. of surgeries (x100)	-5.23*** ± 1.94	-0.68 ± 10.58	-73.19 ± 0.73	-44.36 ± 55.59	-50.95 ± 62.07
Process/structure	-31.89 ± 47.95	120.70** ± 57.01	-15.31 ± 208.29	35.18 ± 132.54	
No chain affiliation	*Reference*	*Reference*	*Reference*	*Reference*	*Reference*
Chain affiliation	-6.23 ± 44.37	-22.88 ± 45.17	30.89 ± 258.90	97.83 ± 253.74	60.16 ± 175.05

Abbreviations: GH, general hospital; ITC, independent treatment centre; THR, total hip replacement; TKR, total knee replacement; CTS, carpal tunnel syndrome; ACL, anterior cruciate ligament. Values are presented as coefficient ± clustered standard error. *** *P*  < .01, ** *P*  < .05.

#### Insurance Contracts

No relationship was detected between the number of insurance contracts for 2018 and quality data of ITCs in 2017 (Table 4). This suggests that insurance contracts are independent of quality of care within the ITC sector.

**Table 4:** Four Ordered Logistic Regression Models on the Relation Between Insurance Contracts of ITCs Concluded in 2018 and Quality in 2017

	**Number of Insurance Contracts [0-4]**
**Model 3**	
Cataract model – fraction ≤1 dioptre of target refraction	10.17 ± 13.06 (30)
Cataract model – fraction improved visual acuity ≥1 line	1.65 ± 3.33 (30)
CTS model – fraction infection	-97.44 ± 98.48 (20)
ACL injury model – fraction revision	82.05 ± 65.11 (14)

Abbreviations: ITCs, independent treatment centres; CTS, carpal tunnel syndrome; ACL, anterior cruciate ligament. Values are presented as coefficient ± clustered standard error (n).

### Robustness Checks

Robustness checks are displayed in Tables S3 to S7. Diverging from the 2017 results, no statistically significant differences in quality between ITCs and GHs were found in 2016 (Table S4, Model 1). Fewer revisions after THR occurred in 2016 compared to 2017, especially for ITCs (Table 1 and Table S3). The corrected zero-or-one inflated models show that the inferior performance of ITCs on THR compared to GHs in 2017 (Table 2), was not found in 2016 (Table S4, Model 1). Also, the significant relationship between ACL and type of facility disappeared in 2016 (Table S4, Model 1). Furthermore, our findings from 2017 indicate a volume-quality relationship for CTS, but this relationship disappeared in 2016; and, vice versa, no volume-quality relationship was found for TKR in 2017 (Table 2), but there was such a relationship in 2016 (Table S4, Model 1). In addition, the process and structure indicators are still associated with better performance on cataract care, but in 2016, the process and structure indicators are related to the other cataract quality measure (ie, postoperative improved visual acuity) instead of the postoperative dioptre of target. These findings reaffirm that no robust quality differences could be detected between ITCs and GHs.

When speciality and academic hospitals are included, the corrected quality outcomes change slightly in favour of ITCs (Table S5). For example, in contrast to Table 2, ITCs perform significantly better than general, specialty and academic hospitals on postoperative dioptre of target refraction after cataract surgery. One of the explanations for this disparity is that academic hospitals treat a different patient-base when performing cataract surgery.^30^ This might suggest that the exclusion of ocular comorbidity does not fully correct for case-mix differences between the different types of providers. Furthermore, the significant relationship between the probability of having revisions within one year after THR and type of provider disappears. This implies that academic hospitals (this analysis does not include specialty hospitals) have similar quality performances as ITCs. Hence, it seems to be that particularly GHs performed well on this measure in 2017. To conclude, facility type remained unrelated to price differences after inclusion of speciality- and academic hospitals (Table S6).

The final robustness check, which includes the outliers with Cook’s Distance values above 0.85, illustrates that the 2 outliers have a substantial impact on the results (Table S7). ITCs become associated with a higher chance of revisions for TKR, while in the main model (Table 2), we did not find such a relationship. In addition, the relationship between facility type and ACL care disappeared in the model when the outlier was included.

## Discussion

This study compared ITCs with GHs on quality and price, and expected ITCs to provide better quality at a lower price – based on the focus factory theory. However, quality differences were inconsistent over different medical procedures and over time. ITCs needed fewer revision surgeries after ACL surgery. In contrast, revision surgery after THR was performed more often in ITCs than in GHs. However, these differences did not persist when we performed the robustness checks. No significant relationship was found between lists prices and facility type (ie, ITCs and GHs). This is relevant for patients with a restrictive-provider plan, as they may need to pay the difference between the list price and 75% of the mean contracted price when they decide to visit a non-contracted provider.^17^ Furthermore, the underlying factors did not demonstrate a clear relationship with quality or price over the different medical procedures. This indicates that ITCs may not strategically compete for patients by offering lower prices or better clinical outcomes.

These empirical findings are in line with international empirical literature, which illustrated inconsistent quality differences between ITCs and hospitals.^30,35-38^ Recent evidence from the Netherlands identified quality differences between ITCs and GHs in providing cataract care.^30^ This study found that ITCs scored significantly higher on patient satisfaction compared to GHs, but patient reported outcomes were similar.^30^ Empirical evidence from England points towards better clinical outcomes after THR and TKR in ITCs compared to NHS providers. However, differences were small and the authors did not fully adjust for case-mix differences.^35^ Browne et al found slightly better outcomes in patients treated in ITCs, but the authors state that ‘such differences were minor and unlikely to be clinically significant.’^36^ In the United States, Chukmaitov et al found no difference in quality of ambulatory surgery centres and hospitals,^38^ while Hollingsworth et al found fewer complications after urological surgery for ambulatory surgery centres.^39^ The inconsistent findings on clinical quality outcomes could be caused by ITCs that focus more on aspects such as patients’ experiences and satisfaction.^40,41^ In line with research on hospital chain affiliation in the United States, our study found no indications that chain affiliation improves quality of care.^42^

We found no overall differences in list prices between ITCs and GHs. The Dutch Health Care Authority (NZa) found approximately 10%-15% lower contracted prices across the board for ITCs compared to GHs,^11^ and another, more recent empirical study finds that ITCs offer 8% lower contracted prices than GHs for cataract care.^30^ A lower contracted price could indicate that ITCs have less bargaining power vis-à-vis insurers. This reasoning is supported by existing literature, which found lower profit margins for ITCs compared to GHs.^43^ Alternatively, lower contracted prices could reflect ITCs being more efficient. However, hospitals may be equally efficient, but may use higher margins on procedures that can be standardised easily to cross-subsidize more loss-making procedures. This requires additional research.

Similar to the quality outcome measures, the volume-quality relationship and the relationship between quality and the process and structure indicators vary over time. We found that facilities with more CTS surgeries scored better on clinical outcomes (ie, less postoperative infections) in 2017, but not in 2016. In contrast, there was a significant volume-quality relationship for THR in 2016, but this relationship was not detected in 2017. Furthermore, we did not find a volume-quality relationship for the other treatments. The volume-quality relationship has been demonstrated in previous research on high-risk surgical procedures in hospitals,^44-49^ but is less studied for low-risk surgical outpatient procedures.^50,51^ Previous research has demonstrated that high-volume hospitals provided better quality of care for low-risk invasive treatments.^50,52-54^ One contribution from the United States and the Netherlands shows that the volume-quality relationship also persist within the ITC sector, however, this relationship appears to be weaker.^51,55^ Different from the previous studies, our results do not indicate that a volume-quality relationship exists for the 5 treatments included in this study.

Lastly, we did not find convincing evidence that healthcare insurers selectively contract ITCs based on quality. Therefore, ITCs may not obtain a competitive advantage when outperforming on quality. This goes against the premise of the regulated competition system that high quality gets rewarded through selective contracting. Studies on the relation between quality and selective contracting in the managed competition sector in the Netherlands are limited, and show mixed results.^56-58^ Studies on price competition in the Dutch hospital-sector also show limited responsiveness of insurers to price differences.^14,15,59^ One study found an increase in total costs of inpatient DRGs after the introduction of market-based price competition, but a decrease in total costs of outpatient DRGs.^14^ Heijink et al detected no decrease in cataract prices after the introduction of regulated competition, and insurers did not selectively contract hospitals on cataract care.^15^ The role of quality in negotiations between insurers and providers seems to be limited.^59^

## Limitations

Despite the uniqueness of the quality dataset – which contains quality data of both GHs and ITCs for multiple treatments – some data limitations need to be taken into account when interpreting our results. Firstly, we cannot exclude the possibility of unobserved confounders, such as remaining case-mix differences. The models that include specialty and academic hospitals indicate concerns of such sort. International evidence allude to concerns of ‘cherry-picking’ behaviour among ITCs, which means that ITCs treat less complex patients compared to GHs.^60,61^ However, some studies indicate that these case-mix differences are not that pronounced.^30,62^ We could not control for case-mix when assessing CTS and ACL surgeries. Case-mix could potentially drive quality outcomes for CTS treatments.^63^ For instance, ITCs might treat less CTS patients with diabetes compared to GHs,^64^ but differences in postoperative infections between diabetic and non-diabetic patients are not necessarily present.^65^ One risk factor for postoperative complications for ACL surgery is if the patient needs inpatient admission following ACL surgery (ie, overnight stays).^66,67^ This is highly influenced by the type of anaesthesia; regional anaesthesia provides more same-day discharges, while general anaesthesia often requires inpatient admission.^68,69^ It is unclear if the choice of anaesthesia between ITCs and GHs significantly differs – most ITCs and GHs offer both options (ie, same-day and overnight stay). Insufficient case-mix correction could lead to unjustifiable lower quality in hospitals due to more highly-complex surgeries. Even if case-mix differences bias our outcome results towards higher quality for ITCs as a result of ‘cherry-picking,’ the absence of quality differences indicates that remaining case-mix differences may play a limited role, or that absence of quality differences is a conservative conclusion.

Secondly, despite a legal mandate for providers to report their annual quality data to the Dutch National Health Care Institute, some providers did not report their quality data. This could introduce selection bias. Also, one large ITC chain aggregated location-specific outcomes, and had to be excluded. We checked if the observations regarding quality and price of this ITC chain were significantly different from the other observations. We found that these observations were less than one z-score removed from the overall mean. Therefore, we argue that the possibility that the exclusion of this chain will bias our results is limited. To check the completeness of the data, we compared ITCs included in our dataset to all healthcare providers featured on the website of the Dutch Patients Association (ZorgkaartNederland.nl): a tool for patients to choose between healthcare providers. The vast majority of ITCs were present in our dataset – depending on the type of treatment – ranging from 74% to 97%. This indicates that our data covers most of the ITC market, but selection bias could not be ruled out. Again, this suggests that the absence of higher quality in ITCs is a conservative conclusion.

Thirdly, list prices might not reflect real prices, as contracted prices are generally lower than list prices, especially for ITCs. Furthermore, it is unclear if list prices are actually used to inform out-of-network patients. For example, legislation prevents prices of out-of-network care to form a major barrier in patient choice: for patients visiting out-of-network providers at least 75% of the average contracted market price must be compensated by insurers. If 75% of the mean market price would be sufficient to cover marginal costs, out-of-network patients may not be charged any out-of-pocket costs, and the list prices lose their informational value. More research is needed to assess if and how list prices are actually used in practice.

Other limitations include self-reporting, small sample size and ITC physicians working in GHs. Firstly, quality data was self-reported by ITCs and GHs, which could result in positive misreporting (ie, desirable answers). Secondly, our findings are based on a relatively small sample size, which limit the ability to detect small differences. In addition, results from small sample sizes are more susceptible to outliers. This was also demonstrated in the robustness checks. Thirdly, a report from 2013 states that at least 96 ITC physicians (divided over a total of 313 ITCs) also worked as a physician at a GH.^18^ However, the available data did not allow us to correct for physicians that work in both GHs and ITCs. These physicians could transfer knowledge and experience between GHs and ITCs, reducing quality differences between facilities types.

### Implications

Our study contributes to the understanding of how ITCs perform compared to GHs on quality of care, price, and how effective selective contracting is, with regard to quality of care, within the Dutch ITC sector. The ITC sector has become more prominent in many healthcare systems and the need for ambulatory care is likely to grow in the near future, with an increasingly ageing population that will further intensify the demand on, for instance, ophthalmological and orthopaedic care.^70,71^

Despite its limitations, our findings could be of interest to various stakeholders. Firstly, health insurers may want to utilise this information in strategic contracting. We found that ITCs are less often contracted than GHs. From a quality perspective, ITCs do not seem to outperform GHs. Furthermore, while we found no differences in list prices, other studies have shown that contracted prices were lower for ITCs compared to GHs.^11,30^ Thus, reallocating low invasive care to ITCs could still be attractive for health insurers from a cost perspective. Although ITCs and GHs on average perform similarly, substantial practice variation in quality may justify more selective contracting on quality. This could also incentivise both ITCs and GHs to invest in quality. Creating more transparency in healthcare costs and prices is warranted in order to study the economic effects of ITCs. Additionally, transparency could empower patients to make better-informed decisions and lower healthcare costs by creating a more efficient and competitive system.^72^ Enhancing and improving open data in healthcare systems to monitor the performance of different types of providers has the potential to greatly improve the efficiency of the healthcare system. Only with better case-mix adjustments can we assess if specialty and academic hospitals are value-adding entities, and if it is more efficient for some patients to be treated in GHs and/or ITCs. Once those improvements are realised, the ITC sector has the potential to play a more prominent role in the provision of elective care and can potentially contribute to the financial sustainability of the Dutch healthcare system.

## Acknowledgements

We thank all healthcare facilities that provided us with data and information for this study. Our gratitude goes out to the reviewers for their time and valuable comments which improved the quality of our manuscript.

## Ethical issues

Not applicable. We used publicly available data on hospitals and independent treatment centres, which we anonymised. No patient level data was used in this study.

## Competing interests

Authors declare that they have no competing interests.

## Authors’ contributions

ADMT, FMK, NWS and PPTJ were responsible for the research design. ADMT and FMK were responsible for the data analyses and the writing. NWS and PPTJ contributed to the drafting of the manuscript. PPTJ and FMK initiated the study. All authors have read the manuscript and approved for submission.

## Authors’ affiliations

^1^IQ healthcare, Radboud University and Medical Center, Nijmegen, The Netherlands. ^2^Ministry of Health, Welfare and Sport, The Hague, The Netherlands.

## Supplementary files


Supplementary file 1 contains Figure S1 and Tables S1-S7.

## Key messages

**Implications for policy makers**

- Based on the results of this study, independent treatment centres (ITCs) do not seem to deliver higher quality of care compared to general hospitals (GHs). Therefore, it seems to be undesirable to actively reallocate care from GHs to ITCs, solely based on quality arguments.
- No significant relationship between list prices and type of provider (ie, ITCs and GHs) was found. This could have implications for patients with a restricted healthcare provider plan and those who choose a non-contracted healthcare provider.
- Contracting is not related to quality within the ITC sector. The substantial practice variation in quality of care may justify more evidence-based contracting within both the ITC and the GH markets.

**Implications for the public**

Our study found considerable variation in terms of quality outcomes and prices between healthcare facilities – both general hospitals (GHs) and independent treatment centres (ITCs). Patients should take quality and list price variations into account when they have a restricted provider plan, and when choosing a suitable healthcare provider. However, this study concluded that these patients do not need to prefer ITCs over GHs in terms of quality or price.
