# Supplementary material for: Independent Treatment Centres Are Not a Guarantee for High Quality and Low Healthcare Prices in The Netherlands – A Study of 5 Elective Surgeries
Source: Int J Health Policy Manag. 2020 Jan 7;9(9):380–9. doi: 10.15171/ijhpm.2019.144 (PMC7557426; doi:10.15171/ijhpm.2019.144)
Supplement: Supplementary file 1 — contains Figure S1 and Tables S1-S7. [file ijhpm-9-380-s001.pdf]

Supplementary file 1

Table S1. Process and Structure Indicators 2017

	Cataract		CTS		TKR		THR	
	ITC	GH	ITC	GH	ITC	GH	ITC	GH
Multiple appointments are by default scheduled on the same day	0.63 ± 0.49 (32)	0.78 ± 0.41 (65)	0.78 ± 0.42 (23)	0.91 ± 0.28 (70)	0.40 ± 0.51 (15)	0.90 ± 0.30 (69)	0.46 ± 0.52 (13)	0.90 ± 0.30 (69)
Availability of extra services[1]			0.25 ± 0.15 (24)	0.27 ± 0.24 (70)	0.20 ± 0.14 (15)	0.31 ± 0.23 (69)	0.24 ± 0.16 (13)	0.32 ± 0.23 (69)
The use of decision aid					0.60 ± 0.51 (15)	0.69 ± 0.47 (68)	0.62 ± 0.51 (13)	0.68 ± 0.47 (68)
Range of anaesthetics offered[2]					0.77 ± 0.10 (15)	0.96 ± 0.10 (69)		
Patients are always asked which anaesthetic method they prefer	0.42 ± 0.50 (31)	0.94 ± 0.24 (65)						
Reachable by email or telephone after surgery	1.00 ± 0.00 (32)	1.00 ± 0.00 (65)						
Uses the quality registration system of the professional ophthalmology association	0.97 ± 0.18 (31)	1.00 ± 0.00 (65)						
Patients see surgeon before anaesthesia	0.97 ± 0.18 (32)	1.00 ± 0.00 (65)						
Patients can choose between lenses	1.00 ± 0.00 (32)	0.95 ± 0.21 (64)						
Range of lenses offered[3]	0.84 ± 0.24 (32)	0.70 ± 0.31 (65)						
Examination of both hands on the same day			0.68 ± 0.48 (22)	0.73 ± 0.45 (70)				
Supporting disciplines available on site[4]			0.32 ± 0.16 (22)	0.54 ± 0.22 (70)				
Multiple appointments are generally scheduled on the same site			0.88 ± 0.34 (24)	0.79 ± 0.41 (70)				
Patient has contact with surgeon within 6 weeks after surgery			0.96 ± 0.19 (22)	0.92 ± 0.17 (69)				
Mean z-value	-0.12 ± 0.38 (32)	0.08 ± 0.35 (65)	-0.13 ± 0.64 (24)	0.05 ± 0.39 (70)	-0.68 ± 0.55 (15)	0.19 ± 0.47 (69)	-0.39 ± 0.65 (13)	0.13 ± 0.57 (69)
Difference (p-value)	p=0.012		p=0.108		p=0.000		p=0.004	

Values are presented as mean ± standard error (n).
Abbreviations: GH = General Hospital; ITC = Independent Treatment Centre; CTS = Carpal Tunnel Syndrome; TKR = Total Knee Replacement; THR = Total Hip Replacement.
1) ie, e-consult, video-consult, evening consult, schedule online appointments, patient can online access their patient record, patient portal.
2) ie, NSAID, opiates, intravenous anaesthesia, nerve blockade, infiltration with local anaesthetics.
3) ie, monofocal lens, monofocal toric lens, bifocal lens, multifocal lens, multifocal toric lens.
4) ie, physiotherapist, occupational therapist, hand therapist, rehabilitation doctor, nurse practitioner, physician assistant, specialised nurse.

Table S2. Zero-or-one inflated beta regression models on quality in relation to facility type (Model 1a), and in relation to the underlying factors (Model 1b), 2017

	Cataract[1]		CTS	TKR	THR	ACL
Dependent variable	Postoperative ≤1 dioptre of target refraction [0-1]	Postoperative improved visual acuity ≥1 line [0-1]	Postoperative infection [0-1]	Revision within 1 year [0-1]	Revision within 1 year [0-1]	Revision within 1 year [0-1]
**Model 1a**	n=94	n=94	n=89	n=78	n=78	n=79
GH	*Reference*	*Reference*	*Reference*	*Reference*	*Reference*	*Reference*
ITC	0.10 ± 0.11	0.13 ± 0.15	0.19 ± 0.18	0.61 ± 0.33	0.75***± 0.17	-0.66** ± 0.28
**Model 1b**	n=94	n=94	n=89	n=79	n=78	n=80
Number of surgeries (x100)	-0.01 ± 0.00	-0.00 ± 0.01	-0.13** ± 0.06	-0.05 ± 0.04	-0.04 ± 0.04	-0.12 ± 0.14
Process / structure	0.43*± 0.25	-0.15 ± 0.24	-0.24*± 0.13	-0.20 ± 0.14	-0.04 ± 0.11	
No chain affiliation	*Reference*	*Reference*	*Reference*	*Reference*	*Reference*	*Reference*
Chain affiliation	-0.05 ± 0.14	-0.24 ± 0.16	0.34* ± 0.18	0.09 ± 0.17	0.09 ± 0.15	-0.09 ± 0.20

Values are presented as coefficient ± clustered standard error.
Abbreviations: GH = General Hospital; ITC = Independent Treatment Centre; CTS = Carpal Tunnel Syndrome; TKR = Total Knee Replacement; THR = Total Hip Replacement; ACL = Anterior Cruciate Ligament injury.
1) The dependent variables of the cataract models are positively framed (one-inflated beta regressions), where the others are negatively framed (zero-inflated beta regressions).
*** p<0.01, ** p<0.05, * p<0.10

Figure S1. Residual plots of the OLS regression models with price as outcome variable, 2017.

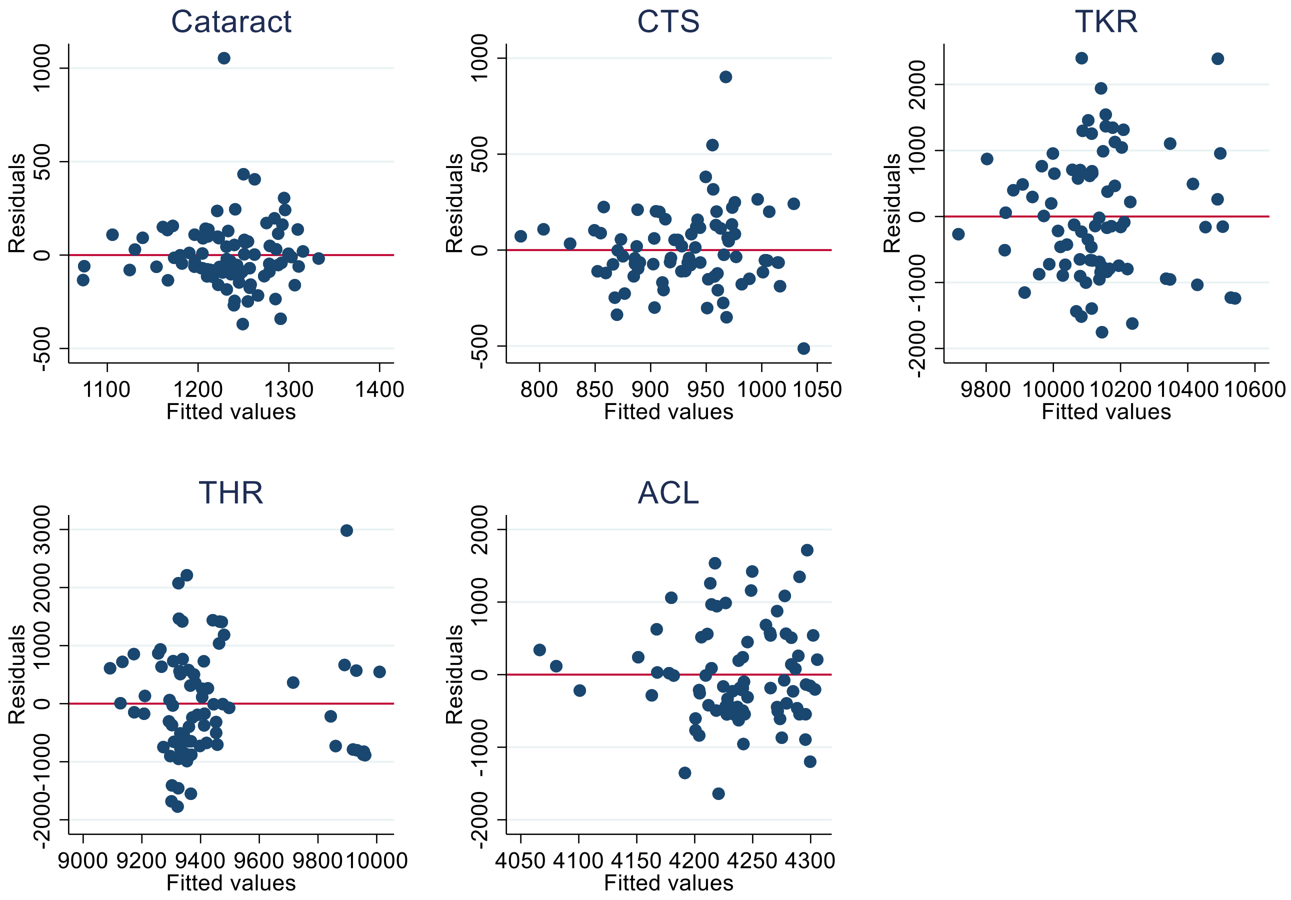

Table S3. Summary statistics 2016

	Cataract		CTS		TKR		THR		ACL	
	**ITC**	**GH**	**ITC**	**GH**	**ITC**	**GH**	**ITC**	**GH**	**ITC**	**GH**
**Quality indicators**										
Postoperative ≤1 dioptre of target refraction [%]	93.73 ± 7.27 (24)	94.00 ± 2.56 (61)								
Postoperative improved visual acuity ≥1 line [%]	88.66 ± 7.58 (24)	90.33 ± 3.83 (61)								
Postoperative infection within 30 days [%]			0.16 ± 0.26 (17)	0.32 ± 0.47 (70)						
Revision within 1 year [%]					1.23 ± 0.89 (8)	0.98 ± 0.71 (68)	1.17 ± 1.23 (6)	1.59 ± 0.93 (68)	2.68 ± 3.49 (13)	3.24 ± 1.97 (68)
Process and structure measure [mean dummies]	0.81 ± 0.10 (26)	0.91 ± 0.10 (65)	0.72 ± 0.21 (20)	0.77 ± 0.15 (70)	0.46 ± 0.22 (10)	0.68 ± 0.17 (69)	0.34 ± 0.25 (7)	0.60 ± 0.21 (69)		
Process and structure measure [index]	-0.21 ± 0.40 (26)	0.06 ± 0.42 (65)	-0.19 ± 0.63 (20)	0.03 ± 0.43 (70)	-0.79 ± 0.64 (10)	0.08 ± 0.50 (69)	-0.74 ± 0.81 (7)	-0.00 ± 0.59 (69)		
**Volume indicators**										
Surgeries [n]	1,177.88 ± 675.35 (25)	1,894.16 ± 928.59 (64)	122.05 ± 162.06 (19)	365.06 ± 194.18 (69)	253.20 ± 243.44 (10)	298.55 ± 136.60 (69)	147.14 ± 121.65 (7)	367.36 ± 191.31 (69)	153.71 ± 148.61 (14)	82.92 ± 69.34 (68)
Chain affiliation	0.54 ± 0.51 (26)	0.54 ± 0.50 (65)	0.50 ± 0.51 (20)	0.53 ± 0.50 (70)	0.70 ± 0.48 (10)	0.52 ± 0.50 (69)	0.57 ± 0.53 (7)	0.52 ± 0.50 (69)	0.64 ± 0.50 (14)	0.53 ± 0.50 (68)

Values are presented as mean ± standard error (n).
Abbreviations: GH = General Hospital; ITC = Independent Treatment Centre; CTS = Carpal Tunnel Syndrome; TKR = Total Knee Replacement; THR = Total Hip Replacement; ACL = Anterior Cruciate Ligament injury.

Table S4. The relationship between quality and facility type (ie, ITC versus GH), 2016

	**Cataract[1]**		**CTS**	**TKR**	**THR**	**ACL**
Dependent variable	Postoperative ≤1 dioptre of target refraction [0-1]	Postoperative improved visual acuity ≥1 line [0-1]	Postoperative infection [0-1]	Revision within 1 year [0-1]	Revision within 1 year [0-1]	Revision within 1 year [0-1]
**Model 1a**	n=84	n=85	n=87	n=76	n=74	n=80
GH	*Reference*	*Reference*	*Reference*	*Reference*	*Reference*	*Reference*
ITC	0.21 ± 0.13	-0.10 ± 0.15	-0.11 ± 0.10	0.14 ± 0.19	0.01 ± 0.17	-0.41±0.22
**Model 1b**	n=85	n=85	n=86	n=76	n=74	n=81
Number of surgeries (x100)	-0.02*** ± 0.01	-0.01 ± 0.01	-0.00 ± 0.05	-0.09** ± 0.04	0.03 ± 0.03	-0.10 ± 0.09
Process / structure	0.17 ± 0.16	0.27** ± 0.13	-0.31 ± 0.19	-0.04 ± 0.08	0.04 ± 0.10	
No chain affiliation	*Reference*	*Reference*	*Reference*	*Reference*	*Reference*	*Reference*
Chain affiliation	0.15 ± 0.14	-0.02 ± 0.14	0.16 ± 0.17	-0.04 ± 0.12	-0.19 ± 0.12	0.02 ± 0.17
**Model 1**	n=84	n=85	n=86	n=76	n=74	n=81
GH	*Reference*	*Reference*	*Reference*	*Reference*	*Reference*	*Reference*
ITC	0.18 ± 0.13	-0.12 ± 0.16	-0.10 ± 0.09	0.24 ± 0.24	0.06 ± 0.16	-0.17 ± 0.31
Number of surgeries (x100)	-0.01 ± 0.01	-0.01 ± 0.01	-0.01 ± 0.05	-0.10** ± 0.04	0.03 ± 0.03	-0.07 ± 0.10
Process / structure	0.19 ± 0.16	0.25** ± 0.12	-0.30 ± 0.19	0.04 ± 0.14	0.05 ± 0.11	
No chain affiliation	*Reference*	*Reference*	*Reference*	*Reference*	*Reference*	*Reference*
Chain affiliation	0.06 ± 0.13	-0.00 ± 0.15	0.17 ± 0.17	-0.04 ± 0.12	-0.19 ± 0.12	0.02 ± 0.17

Values are presented as coefficient ± clustered standard error.
Abbreviations: GH = General Hospital; ITC = Independent Treatment Centre; CTS = Carpal Tunnel Syndrome; TKR = Total Knee Replacement; THR = Total Hip Replacement; ACL = Anterior Cruciate Ligament injury.
1) The dependent variables of the cataract models are positively framed (one-inflated beta regressions), where the others are negatively framed (zero-inflated beta regressions).
*** p<0.01, ** p<0.05

Table S5. Zero-or-one inflated beta regression models on quality in relation to facility type (ie, ITCs versus GHs plus specialist and academic hospitals), 2017

	**Cataract**[1]		**CTS**	**TKR**	**THR**	**ACL**
Dependent variable	Postoperative ≤1 dioptre of target refraction [0-1]	Postoperative improved visual acuity ≥1 line [0-1]	Postoperative infection [0-1]	Revision within 1 year [0-1]	Revision within 1 year [0-1]	Revision within 1 year [0-1]
**Model 1a**	n=103	n=103	n=95	n=89	n=86	n=89
GHs	*Reference*	*Reference*	*Reference*	*Reference*	*Reference*	*Reference*
ITC	0.16 ± 0.10	0.14 ± 0.14	0.07 ± 0.17	0.51 ± 0.32	0.66*** ± 0.16	-0.69** ± 0.28
**Model 1**	n=103	n=103	n=95	n=89	n=86	n=89
GHs	*Reference*	*Reference*	*Reference*	*Reference*	*Reference*	*Reference*
ITC	0.35*** ± 0.12	0.08 ± 0.14	-0.19 ± 0.14	0.37 ± 0.73	0.54 ± 0.31	-0.76*** ± 0.29
Number of surgeries (x 100)	0.01 ± 0.01	-0.01 ± 0.00	-0.19*** ± 0.07	-0.11** ± 0.04	-0.07 ± 0.04	0.04 ± 0.11
Process / structure	0.21 ± 0.19	-0.11 ± 0.16	-0.11 ± 0.19	0.02 ± 0.16	0.11 ± 0.12	
No chain affiliation	*Reference*	*Reference*	*Reference*	*Reference*	*Reference*	*Reference*
Chain affiliation	0.30** ± 0.12	-0.01 ± 0.15	0.22 ± 0.18	-0.17 ± 0.38	-0.34 ± 0.22	-0.18 ± 0.26

Values are presented as coefficient ± clustered standard error.
Abbreviations: GHs = General Hospitals, Academic Hospitals and Specialist Hospitals; ITC = Independent Treatment Centre; CTS = Carpal Tunnel Syndrome; TKR = Total Knee Replacement; THR = Total Hip Replacement; ACL = Anterior Cruciate Ligament injury.
1) The dependent variables of the cataract models are positively framed (one-inflated beta regressions), where the others are negatively framed (zero-inflated beta regressions).
*** p<0.01, ** p<0.05

Table S6. Relation price and facility type (ie, ITCs versus Ghs plus specialist and academic hospitals), 2017

	Cataract	CTS	TKR	THR	ACL
Dependent variable	List price surgery [€]	List price surgery [€]	List price surgery [€]	List price surgery [€]	List price surgery [€]
**Model 2**	n=103	n=93	n=92	n=88	n=90
GHs	*Reference*	*Reference*	*Reference*	*Reference*	*Reference*
ITC	-64.13 ± 40.15	45.22 ± 53.99	2.45 ± 526.06	252.45 ± 417.62	111.54 ± 188.31
Number of Surgeries (x 100)	-4.37*** ± 1.47	-2.55 ± 10.34	-57.64 ± 68.01	-62.42 ± 52.70	-53.86 ± 83.84
Process / structure	-16.38 ± 41.52	108.29 ± 55.39	-117.57 ± 228.85	-53.39 ± 139.98	
No chain affiliation	*Reference*	*Reference*	*Reference*	*Reference*	*Reference*
Chain affiliation	-27.35 ± 44.32	-27.17 ± 42.72	-347.00 ± 306.02	-373.49 ± 314.00	448.89 ± 337.32

Values are presented as coefficient ± clustered standard error.
Abbreviations: GHs = General Hospitals, Academic Hospitals and Specialist Hospitals; ITC = Independent Treatment Centre; CTS = Carpal Tunnel Syndrome; TKR = Total Knee Replacement; THR = Total Hip Replacement; ACL = Anterior Cruciate Ligament injury.
*** $p<0.01$

Table S7. ***Quality in relation to facility type with outliers, 2017***

	**TKR**	**ACL**
Dependent variable	Revision within 1 year [0-1]	Revision within 1 year [0-1]
**Model 1a**	n=79	n=80
GH	*Reference*	*Reference*
ITC	0.79** ± 0.37	-0.47 ± 0.34
**Model 1**	n=79	n=80
GH	*Reference*	*Reference*
ITC	0.94 ± 0.50	-0.41 ± 0.37
Number of surgeries (x100)	-0.03 ± 0.05	-0.03 ± 0.13
Process / structure	0.10 ± 0.19	
No chain affiliation	*Reference*	*Reference*
Chain affiliation	0.15 ± 0.15	0.11 ± 0.19

Values are presented as coefficient ± clustered standard error.
Abbreviations: GH = General Hospital; ITC = Independent Treatment Centre; TKR = Total Knee Replacement; ACL = Anterior Cruciate Ligament injury.
** p<0.05
